# Efficacy of Coil Embolization in Small, Anterior Circulation Aneurysms in Patients Less Than 40 Years Old

**DOI:** 10.3390/jcm13164764

**Published:** 2024-08-13

**Authors:** Dong Sun Park, Hong Gee Roh, Young Il Chun, Yoo Sung Jeon

**Affiliations:** 1 Department of Neurosurgery, Hanyang University Changwon Hanmaeum Hospital, Changwon 51139, Republic of Korea; 2 Department of Radiology, Konkuk University Medical Center, Seoul 05030, Republic of Korea; hgroh@kuh.ac.kr; 3 Department of Neurosurgery, Konkuk University Medical Center, Seoul 05030, Republic of Korea; yichun@kuh.ac.kr

**Keywords:** cerebral aneurysm, endovascular treatment, young patients, recurrence, complications

## Abstract

**Background:** Ruptured and unruptured aneurysms are less common in younger individuals compared to older patients. Endovascular treatment has gained popularity over surgical options in the general population, but surgery remains the primary treatment for younger patients due to concerns about higher recurrence rates with endovascular procedures. **Methods:** This study compared the immediate and long-term outcomes of endovascular treatment in patients under 40 years with those aged 41–60. The study included 239 patients who underwent endovascular treatment for intracranial aneurysms, divided into two age groups: under 40 and 41–60 years. The rates of immediate radiologic outcomes, complications, and recurrence were assessed. **Results:** The results showed successful aneurysm obliteration rates of 70.1% in the younger group and 64.0% in the older group. The complication rates were 1.5% in the younger group and 3.5% in the older group, with the older group experiencing more procedure-related complications, though this difference was not statistically significant. Long-term follow-up revealed recurrence rates of 23.2% in the younger group and 18.2% in the older group, with no significant difference. **Conclusions:** The study suggests that endovascular treatment is as effective and safe for patients under 40 years. Therefore, it may be considered an acceptable first-line treatment for younger patients, aligning its use with that in older populations.

## 1. Introduction

Cerebrovascular disorders, particularly subarachnoid hemorrhage (SAH) and unruptured intracranial aneurysms (UIAs), represent critical medical challenges that predominantly affect individuals in their sixth and seventh decades of life [1,2,3,4]. The incidence of these conditions in younger populations, however, has seen a notable increase, necessitating a reevaluation of existing treatment strategies across different age groups [5]. Traditionally, surgical clipping has been the cornerstone of treatment for young patients with intracranial aneurysms due to its proven long-term efficacy and durability [6,7,8,9,10,11,12]. This clipping ensures a permanent solution, which is crucial considering the potentially extended lifespan of these younger patients [13,14,15,16].

Despite the entrenched preference for surgical clipping among younger patients, the landscape of aneurysm treatment has evolved with the advancement of endovascular techniques [6,7,10,12,13]. Endovascular treatment, such as coiling using balloons or stents and flow-diverters, has gained prominence due to its minimally invasive nature, reduced perioperative risks, and shorter recovery periods [17,18]. These advantages have led to its preferred use in the general population, particularly among older patients [2,3,4,15,19,20,21,22]. Nevertheless, there exists a lingering hesitation to adopt these newer methods in younger demographics, primarily due to concerns about higher recurrence rates and long-term outcomes compared to surgical clipping [13,15,17]. However, the advancements in endovascular techniques over the past decades have introduced alternatives that could potentially match or even surpass the outcomes of surgical clipping in specific contexts [18]. This has prompted a growing interest in exploring the viability of these techniques across different patient demographics, including younger populations.

Given this backdrop, the current study seeks to explore the viability of endovascular treatment as a first-line therapy in patients under the age of 40. By comparing the immediate and long-term treatment outcomes of endovascular treatment in young patients to those observed in older patients (ages 41–60), this study aims to determine whether the less invasive endovascular approach could challenge the prevailing surgical paradigm.

This study will also compare the clinical outcomes between unruptured and ruptured groups, evaluating complication rates, long-term recurrence, and other factors. Additionally, we will examine the limitations of endovascular treatment compared to surgical clipping and the implications for the follow-up period.

The findings are expected to provide a balanced perspective on the choice of treatment modality, tailored to individual patient profiles rather than predominantly age-based criteria. Overall, this research aims to provide a comprehensive understanding of the safety and efficacy of endovascular treatment for cerebral aneurysms in younger patients, potentially influencing future clinical and follow-up guidelines.

## 2. Materials and Methods

**Figure 1:**
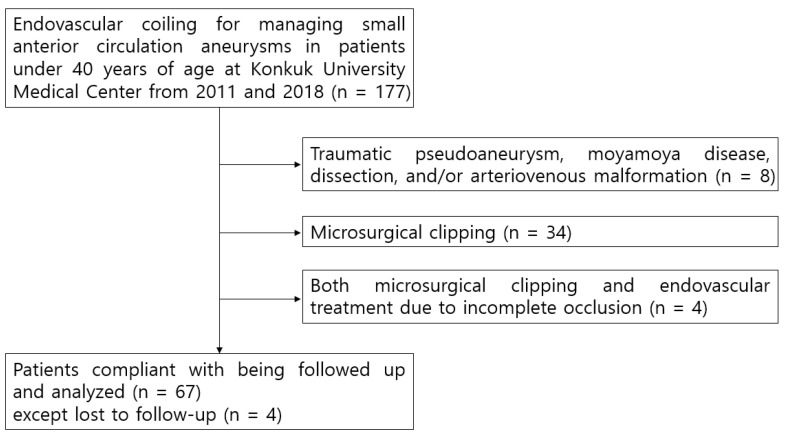
Endovascular coiling for managing small anterior circulation aneurysms in patients under 40 years of age.

Retrospectively recorded data of all aneurysmal SAH and UIA patients during January 2011 and January 2018 at Konkuk University Medical Center were reviewed. Among these, 117 patients less than 40 years of age with anterior circulation aneurysms of less than 10 mm in maximum dome diameter were included in the initial pool. Of these, there were 8 patients with aneurysms and pseudoaneurysms associated with trauma, Moyamoya disease, dissection, and/or arteriovenous malformation; 34 patients who underwent microvascular surgery; 4 patients who underwent both microvascular surgery and endovascular treatment due to incomplete occlusion; and 4 who either expired or were transferred to another center within 1 week after endovascular treatment who were excluded (Figure 1). As such, the final study subject included 67 patients. Data of 158 SAH patients and 126 UIA patients aged between 41 and 60, which is the typical age group for the disease, were also enrolled as the control group. After applying the exclusion criteria as mentioned above, a total of 172 patients were included in the control group.

Our initial evaluation was geared towards a demographical classification of patients based on age group, sex, aneurysm location, and geometry. The initial neurological status at presentation was measured with a Hunt–Hess grade from 1 to 5, with an additional grade 0 for the unruptured aneurysms.

Secondly, immediate angiographic results and procedure-related complications were analyzed. The angiographic results were graded into three groups: “Complete occlusion”, “Residual neck”, and “Residual sac”, depending on the extent of contrast filling within the aneurysm sac and/or neck.

Lastly, all aneurysms were treated using either regular coiling (single or double microcatheter), balloon-assisted coiling, or stent-assisted coiling under general anesthesia, and the long-term radiographic and clinical outcomes were analyzed in a subgroup of patients who had been followed at our center. During the hospitalization period, CT angiography was performed around one week later, and for vasospasm, a continuous infusion of calcium channel blockers was administered. For symptomatic vasospasm, angioplasty (chemical or balloon) was performed. Image follow-up after discharge was carried out routinely with magnetic resonance angiography (MRA), with additional digital subtraction angiography (DSA) when recurrence was suspected. The radiologic outcome was graded by the same grading system used for the immediate results. Recurrence was defined as progressive contrast filling within the coiled aneurysm in the follow-up studies for cases with a follow-up period of at least 6 months. However, the distinction between minor recurrences that were observed and major recurrences that were re-treated depended on the surgeon’s individual opinion. Additionally, the differences between the groups were examined in terms of the need for further surgeries due to complications of SAH, such as acute/chronic hydrocephalus, severe vasospasm, and decompression due to intracranial hemorrhage or brain swelling. The clinical outcome was worked out by measuring the Modified Rankin Scale (mRS) at discharge and after one year.

All values were compared with those of the older-aged control group and statistically analyzed. Furthermore, statistical analysis was conducted on the recurrence rates in both the unruptured and ruptured groups, as well as the relationship between age and recurrence. For the association analyses, the Chi-square test, Fisher’s exact test, and Kappa test were utilized. All analyses were performed at a significance level of *p* < 0.05 using SAS Version 9.4 (SAS Institute Inc., Cary, NC, USA).

All endovascular surgeries were performed within 72 h of admission by 2 vascular- and endovascular-trained neurosurgeons and 1 interventional neuroradiologist. Detachable platinum coils were used in all cases; a stent was used in selected patients with wide-necked aneurysms.

## 3. Results

A total of 67 patients with intracranial aneurysms under the age of 40 were treated using endovascular coil embolization. The mean age of the patients was 35.3 ± 4.6 years (range, 20–40; median, 36), and the male-to-female ratio was 1:1.12 (32 males vs. 36 females). Twenty-three patients (34.3%) presented with unruptured aneurysms, and they were regarded as Hunt–Hess grade 0. Among the rest who presented with SAH, 35 patients (52.2%) were graded within Hunt–Hess 1 and 3, and 9 patients (13.4%) within Hunt–Hess 4 and 5. The mean aneurysm size was 4.9 ± 1.7 mm (range, 2.5–9.8 mm) in maximum dome diameter. Concerning aneurysm location, 29 aneurysms (43.3%) were located at the anterior cerebral artery (ACA) and anterior communicating artery (ACoA), 6 (8.9%) at the MCA bifurcation, 21 (31.3%) at the ICA (except the paraclinoid segment), and 11 (16.4%) at the paraclinoid ICA. A control group of 172 intracranial aneurysm patients aged between 41 and 60 was compared, and there were no differences between the two groups with respect to sex, aneurysm location, and the size of the proportion of ruptured aneurysms (Table 1).

All aneurysms were treated using either regular coiling, balloon-assisted coiling, or stent-assisted coiling under general anesthesia. Flow-diverter use was not employed due to insurance issues, as it was only approved in Korea for unruptured aneurysms ≥ 15 mm. Among the followed-up patients, there were 89 in the unruptured group and 104 in the ruptured group, with 19 and 37 patients in the younger age group, respectively. In terms of treatment methods, double-microcatheter or balloon/stent-assisted coiling was used in 58 patients (64%) in the unruptured group, which was significantly higher compared to 41 patients (39.4%) in the ruptured group (*p* < 0.05). Recurrence was significantly lower in the unruptured group, with nine patients (10.1%) experiencing recurrence. Additionally, there was no statistically significant correlation between age and recurrence rate (Table 2).

**Figure 2:**
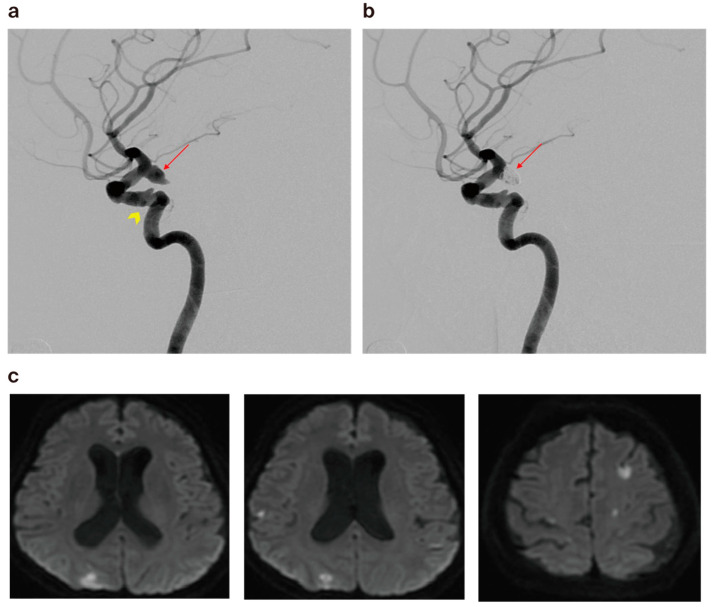
(**a**) A 59-year-old female patient’s cerebral angiography revealed ruptured right posterior communicating artery aneurysm (arrow) and atherosclerotic change in the left cavernous ICA (arrowhead). (**b**) Cerebral angiogram showing minimal residual filling of the neck after coiling (arrow) via the double microcatheter technique. (**c**) Diffusion-weighted imaging obtained 1 day post embolization, revealing multiple tiny acute infarctions.

The initial angiographic results revealed complete occlusion in 47 cases (70.1%), residual neck in 14 cases (20.9%), and residual sac in 6 cases (9.0%). The results in the older-aged control group were 110 cases (64.0%) for complete occlusion, 44 cases (25.6%) for residual neck, and 18 cases (10.5%) for residual sac. There was one case of intra-procedural aneurysm rupture in the study group, while two cases (1.2%) were observed in the control group. There were 6 cases (9.0%) of minor thromboembolism that were either self-resolved or resolved after an intraarterial infusion of glycoprotein IIb/IIIa inhibitor (Tirofiban hydrochloride) in the study group, compared to 12 (7.0%) in the control group. None of these patients were symptomatic. Thromboembolism resulting in the occlusion of a branch with transient neurologic symptoms was identified in four cases (2.3%) in the control group only (Figure 2). There were no procedure-related complications resulting in death. However, the differences between the two groups were not significant in the initial angiographic results (Table 3).

**Figure 3:**
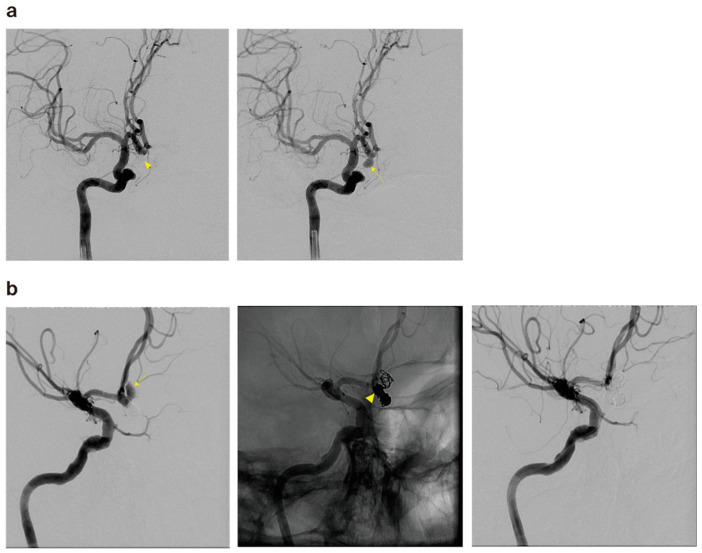
(**a**) The cerebral angiography of a 57-year-old male patient revealed a ruptured right anterior communicating artery aneurysm (arrow) and postoperative angiography after coil embolization (arrowhead). (**b**) The patient presented to the emergency room with SAH at 30 months after the treatment, and cerebral angiography revealed coil compaction and aneurysm regrowth with a newly observed pseudoaneurysm (arrow). Partial coiling was performed on the pseudosac, and compact embolization was performed on the common neck portion (arrowhead).

Additionally, there was one case of rebleeding from a coiled aneurysm in each group, with the younger group experiencing rebleeding at 36 months and the older group at 30 months, both cases being instances of delayed bleeding (Figure 3).

**Figure 4:**
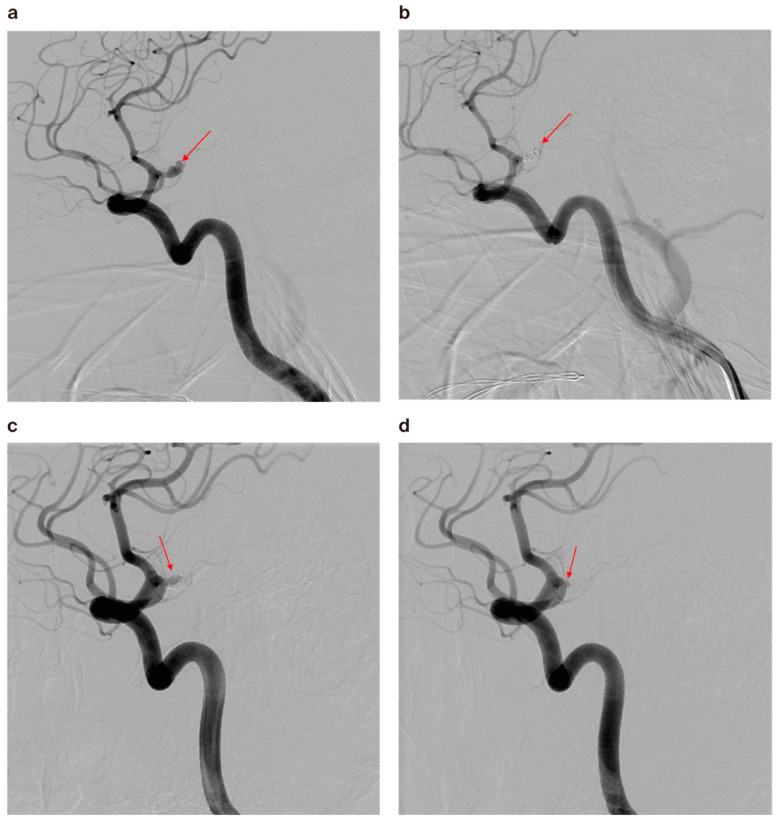
(**a**) A 30-year-old male patient’s cerebral angiography revealed a ruptured right A1 anterior cerebral artery aneurysm (arrow). (**b**) Cerebral angiogram showed nearly complete occlusion after coiling (arrow). (**c**) Follow-up angiography after 6 months revealed major recanalization (arrow). (**d**) Cerebral angiography after retreatment showed minimal residual neck (arrow).

Follow-up MRA and/or DSA data from longer than 6 months after treatment were available in 56 out of 67 (83.6%) aneurysms in the younger group. The same data were available in 137 out of 172 (79.7%) aneurysms in the control group. The follow-up angiography results of the under-40 group revealed complete occlusion in 40 cases (71.4%), residual neck in 11 cases (19.6%), and residual sac in 5 cases (8.9%). The rate of complete occlusion was slightly higher than in the control group (89 out of 137 cases, 65%), but there was no statistical significance (*p* = 0.811, Table 2). Eight cases (14.3%) had minor recurrence and were further observed, whereas five cases (8.9%) showed major recurrence that necessitated prompt retreatment (Figure 4). There was one case of rebleeding from a coiled aneurysm in each group. The recurrence and retreatment rates were slightly higher in the younger than the older group, but the difference was not significant (Table 2).

There were 37 younger patients with ruptured aneurysms and 67 patients in the older group. Craniotomy or decompression was required in six (16.2%) and seven patients (10.4%), respectively. External ventricular drainage (EVD) due to acute IVH was necessary in 8 patients (21.6%) in the younger group and 17 patients (25.3%) in the older group, which was not statistically significant. Ventriculoperitoneal shunt (VPS) for chronic complication hydrocephalus was 5.4% lower in the younger group (Table 2).

Outcome was measured based on mRS at the time of discharge and one year after discharge. In the study group, good outcomes (defined as mRS 0–2) were achieved in 59 out of 67 patients (88.1%) at discharge and in 59 out of 62 patients (95.2%) after 1 year. The proportion of good outcomes in the study group was slightly higher than that of those in the control group (87.2% and 93.4% at discharge and after 1 year, respectively), but the difference failed to prove significant (*p* = 1.000 and 0.764, respectively).

## 4. Discussion

The findings from our study suggest that coil embolization for cerebral aneurysms yields promising clinical outcomes across both younger and control groups, highlighted by low complication rates and no procedure-related fatalities. This efficacy underscores the potential of endovascular treatments to serve as a mainstream therapeutic strategy, challenging the traditional dominance of surgical interventions, particularly in younger patients where surgical clipping has been preferred due to its perceived long-term reliability.

In our analysis, the control group exhibited a slightly higher rate of immediate complications (3.5%) compared to the younger group (1.5%). While these findings could have been anticipated given the control group’s propensity for complex vascular conditions such as tortuosity, advanced atherosclerosis, and stenosis, the difference did not reach statistical significance. This lack of significance, possibly attributed to the small sample size of our study, calls for caution in interpretation and highlights the need for further research involving larger cohorts to better understand the true impact of age on procedural risks.

Moreover, this study addresses the intriguing aspect of aneurysm management concerning recurrence and retreatment rates. Although these rates were slightly higher in younger patients (14.3% and 8.9%, respectively) compared to the control group (12.4% and 5.8%, respectively), they were not statistically significant. These findings contrast with previous studies that reported significantly higher recurrence rates in younger individuals [6,17]. Although not statistically significant, the immediate occlusion rate and the follow-up occlusion rate showed slight improvement in both groups. This suggests that coil embolization can be an effective and viable option even in younger patients. However, it is important to note that our study did not stratify the initial occlusion rates and included both unruptured and ruptured groups, which warrants caution in interpretation. Nonetheless, it is noteworthy that, despite Hyun Sik Kim et al. stating that double-microcatheter or balloon-/stent-assisted coil embolization reduce recurrence rates by increasing the packing ratio compared to single-microcatheter treatment [23], our results showed favorable outcomes in the younger group even with a higher frequency of single-microcatheter use. The complete occlusion rate was slightly higher, and the smaller size of the aneurysms might have contributed to these outcomes. Consequently, these findings suggest that, while traditional surgical clipping might be considered for younger patients due to its perceived long-term durability [24], endovascular treatment could be a safe option with low complication rates and an effective method with low recurrence rates.

Additionally, we compared the unruptured and ruptured groups and obtained results consistent with traditional outcomes [25,26]. The unruptured group had a statistically significantly lower recurrence rate compared to the ruptured group. This may be attributed to the higher proportion of double-microcatheter use or balloon-/stent-assisted coil embolization rather than the frequency of single-microcatheter use. Furthermore, although we did not analyze it, we assume that adequate preoperative preparation, the packing ratio, and the initial occlusion rate may have an impact, and further research on these factors is needed.

Regarding the complications of SAH, we found that the younger group had lower rates of external ventricular drainage, ventriculoperitoneal shunt, and vasospasm cases, except for those requiring craniotomy or decompression. This could be because symptoms related to cerebral edema or herniation occur earlier in younger patients, increasing the likelihood of requiring surgery [27].

Traditionally, clipping has shown better surgical outcomes for anterior circulation aneurysms smaller than 5 mm [13,24]. Giada Toccaceli et al. reported a post-clipping occlusion rate of 96.3% for MCA aneurysms [28]. In our study, the young age group had a total occlusion rate of 91.0%, including residual neck remnants, which is lower compared to that for clipping. The endovascular approach, with the use of newly available devices, appears to enhance the likelihood of achieving a good functional outcome after treatment when compared to surgery [18,28]. Additionally, Zhu, W et al. reported through a prospective study that, while the risk of rebleeding was higher in ruptured cases, the clinical outcomes were better [18,28]. Based on this, while clipping may be clearly effective in preventing rebleeding, the advancements in endovascular technology and superior clinical outcomes suggest that it could be a very good treatment option for younger patients.

However, as our report also indicates, cases of delayed rupture do exist. E. Crobeddu et al. recommended imaging follow-up at 6 months after treatment for ruptured cases, and if stable, follow-up at 3 to 5 years. However, they suggested tailoring the examination schedule based on the individual patient’s condition and aneurysm characteristics [29]. In our cases, there were two instances of delayed rupture, both occurring more than two years post treatment. This highlights the need for caution against the traditional reports suggesting that most recurrences occur within six months, with a decreased likelihood of recurrence thereafter. The delayed rupture cases mentioned above are considered to be de novo aneurysms occurring in a different direction from the original aneurysm. Particularly for ruptured cases, it is recommended to consider MRA examinations annually [29].

In summary, the statistically insignificant differences in complication and recurrence rates across age groups in our study could play a crucial role in re-evaluating treatment paradigms. This supports a more balanced perspective where treatment modality choices are tailored to individual patient profiles rather than predominantly age-based criteria. However, it is important to consider that these conclusions are influenced by the limitations of our study’s small sample size and retrospective design, which may affect the generalizability of the results.

Our data support the safety and efficacy of endovascular treatment across a wide age range, yet the slightly higher recurrence and retreatment rates observed in younger patients underscore the importance of rigorous follow-up regimes. Regular radiological and clinical assessments are paramount for the early detection of recurrences and the management of rebleeding risks, so as to ensure long-term patient safety and treatment success [11]. Future studies should aim to expand on these findings with larger, possibly multicentric cohorts to provide a more robust dataset, thereby refining clinical guidelines for managing cerebral aneurysms in diverse patient populations.

## 5. Limitation

This study was composed of a small cohort, and there may have been sampling errors. The term “recurrence” has been universally applied to contrast filling within the aneurysm without making a distinction between aneurysmal regrowth, coil compaction, and sac recanalization. Being a retrospective study, there was no unified standard for retreatment in recurred aneurysms. Three individual physicians followed their own opinion in deciding retreatment and this may have affected the result. The absence of antiplatelet premedication and the need for promptness in the ruptured cases may affect the procedure time and coil packing density, and hence affect the immediate and long-term results. Furthermore, the location of the aneurysms was not taken into account, and the complexity of the patient groups inevitably introduces statistical limitations. These limitations may be supplemented in further prospectively designed studies.

## 6. Conclusions

Our data showed that endovascular immediate radiologic outcomes, complication rates, and recurrence rates did not differ significantly between the two age groups. Moreover, procedure-related complications were lower in the younger patient group, and the clinical outcomes were also better. Additionally, the recurrence rate was higher in the ruptured group compared to the unruptured group. There were cases of delayed rupture after 2 years, indicating the need for regular long-term follow-up examinations.

We suggest that endovascular treatment is a viable and effective first-line option for both younger and older patients with cerebral aneurysms. Despite the slightly higher recurrence rate in ruptured cases, the overall safety and efficacy of the endovascular approach remain favorable. It is crucial to implement rigorous and long-term follow-up regimes to monitor for potential recurrences and manage rebleeding risks. Future research should focus on larger, multicentric cohorts to further validate these findings and refine clinical guidelines, ensuring that treatment strategies are tailored to individual patient profiles and conditions.

## Figures and Tables

**Table 1:** Baseline characteristics of small anterior circulation aneurysms in patients less than 40 years of age.

		Study Group (20–40 Years)		Control Group (41–60 Years)		*p*
Cases		67		172		
Age	mean ± SD (median)	35.3 ± 4.6	(36)	51.7 ± 5.4	(52)	
Sex	Male–Female	1:1.1		1:1.9		0.054
Presentation	Hunt–Hess					
	0	23	(34.3)	80	(46.5)	0.170
	1~3	35	(52.2)	78	(45.4)	
	4~5	9	(13.4)	14	(8.1)	
Aneurysm						
Size	mean ± SD (range)	4.9 ± 1.7	(2.5~9.8)	5.3 ± 1.8	(1.8~10.0)	
Location	*n* (%)					
	ACA and ACoA	29	(43.3)	62	(36.0)	0.509
	MCA	6	(8.9)	24	(14.0)	
	ICA except paraclinoid	21	(31.3)	63	(36.6)	
	Paraclinoid ICA	11	(16.4)	23	(13.4)	

ACA: anterior cerebral artery; ACoA: anterior communicating artery; MCA: middle cerebral artery; ICA: internal carotid artery; *n*: number of patients.

**Table 2:** Treatment methods and recurrence in unruptured and ruptured aneurysms.

		Unruptured	Ruptured	Study (56)	Control (172)
Patients, *n* (20~40 years, 41~60)		103 (23 + 80)	136 (44 + 92)		
Followed-up patients, *n*		89 (19 + 70)	104 (37 + 67)		
Treatment methods (*n*, %)	Single microcatheter	32 (36.0)	63 (60.6)	31	64
	Double microcatheter	18 (20.2)	25 (24.0)	9	34
	Balloon assisted	9 (10.1)	3 (2.9)	4	8
	Stent assisted	30 (33.7)	13 (12.5)	12	31
Recurrence (*n*, %)		9 (10.1)	29 (27.8)	*p* = 0.028	
Correlation analysis between age and recurrence rate				*p* = 0.434	
Ruptured patients, *n* (%) 20~40 years–41~60 years		37:67			
Complication, *n* (%)	External ventricular drainage	8 (21.6):17 (25.3)			
	Craniotomy or decompression	6 (16.2):7 (10.4)			
	Angioplasty (chemical/balloon)	7 (18.9):13 (19.4)			
	Ventriculoperitoneal shunt	2 (5.4):12 (17.9)			

**Table 3:** Immediate and long-term treatment results of coiling in small anterior circulation aneurysms in patients less than 40 years of age.

		Study Group (20–40 Years)		Control Group (41–60 Years)		*p*-Value
Immediate Occlusion	*n* (%)					
	Complete	47	(70.1)	110	(64.0)	0.661
	Residual Neck	14	(20.9)	44	(25.6)	
	Residual Sac	6	(9.0)	18	(10.5)	
Procedure-Related Complications						
	Aneurysm Rupture	1	(1.5)	2	(1.2)	1.000
	Thromboembolism	0		4	(2.3)	
	Death	0		0		
Follow-up						
	Cases	56		137		
	Interval (months)	27.5 ± 20.8		24.5 ± 17.9		
Follow-up Occlusion						
	Complete	40	(71.4)	89	(65.0)	0.811
	Residual Neck	11	(19.6)	35	(25.5)	
	Residual Sac	5	(8.9)	13	(9.5)	
Recurrence						
	Observation	8	(14.3)	17	(12.4)	0.739
	Retreatment	5	(8.9)	8	(5.8)	0.578
Rebleeding from Coiled Aneurysm						
		1	(1.8)	1	(0.7)	1.000
Clinical Outcome	mRS 0~2					
	At Discharge	59	(88.1)	150	(87.2)	1.000
	After 1 Year	59	(95.2)	156	(93.4)	0.764
	Expired	3	(4.5)	7	(4.0)	

## Data Availability

The data that support the findings of this study are available on reasonable request from the corresponding author.
